# Supplementary material for: Interfacial Ru/RuOx heterostructures on carbon support regulate selectivity in lignin hydrodeoxygenation
Source: Nat Commun. 2026 Apr 11;17:4878. doi: 10.1038/s41467-026-71394-z (PMC13230832; doi:10.1038/s41467-026-71394-z)
Supplement: Supplementary file 1 — Supplementary Information [file 41467_2026_71394_MOESM1_ESM.pdf]

Supplementary information

Interfacial Ru/RuO_x Heterostructures on Carbon Support Regulate Selectivity in Lignin Hydrodeoxygenation

Hongfei Ma, Chen Chen, Guoyan Ma, Tian Ouyang, Hao Zhang, Qixian Wang, Wenjing Wei, Yifan Li, Yi Cui, De Chen

1. Materials and experimental methods

1.1 Materials

The corncob residue was provided by Shandong Longlive Biotechnology Co., Ltd. It is extracted from the residue of functional sugar prepared from corncobs. This type of lignin was assigned as the waste, with a price of only ~200 CNY per ton. It contains 91% wt% of lignin. The elemental analysis shows that the weight concentrations of C, H, O, N, and others are 61.29%, 6.69%, 29.61%, 0.98%, and 1.43, respectively. [1]

Beechwood biomass was provided by Dansk Traemel, with the elemental analysis of C: 49.9 wt%, H: 6 wt%, O: 43.5 wt%, ash: 0.6 %.

n-Dodecane (99%) was purchased from Sigma-Aldrich and was directly used without any pretreatment. Ru supported on activated carbon (Ru/AC) and γ-Al_2O_3 (Ru/Al_2O_3) was purchased from Sigma-Aldrich.

1.2 Catalyst preparation

Carbon nanofibers (CNF) used in this work were prepared by the decomposition of CO over Fe catalyst. In brief, 0.1 g iron oxide (Fe_3O_4) was reduced by 10% H_2/Ar at 600 °C for 5 hours at a packed bed reactor. Then introduce CO/H_2 (4/1) to the reactor for 12 hours to grow and synthesize CNF. The obtained CNF was washed in 70% nitric acid at 90 °C for 5 hours. Then followed by drying at 100 °C overnight. 5 wt% Ru was impregnated on the CNF by using $RuCl_3$ as the precursor and dried at 100 °C overnight. Then, the obtained powders were reduced by 10% H_2/Ar at 300 °C for 5 hours.

The CNF after acid treatment was calcinated at 700 °C (or 1000 °C) and stayed at Ar (100 ml/min) for 3 hours to remove the oxygen functional species, and it was denoted CNF-700 (and

CNF-1000). The same procedure was employed to prepare the Ru catalyst, denoted Ru/CNF-700 and Ru/CNF-1000.

1.3 Catalyst characterization

1.3.1 Physical structure of the catalysts

XRD profiles were recorded with a Bruker D8 Davinci X-ray diffractometer (Bruker Nano GmbH, Berlin, Germany), using Cu Ka1 (0.154 nm) wavelength. The high-resolution transmission electron microscopy (HR-TEM) was performed on a JEOL JSM-2100F system.

The high-angle annular dark-field scanning transmission electron microscopy (HAADF-STEM) was conducted on the JEM-F200 at an acceleration voltage of 200 kV.

The specific surface areas were measured on a TriStar 3020 instrument at liquid nitrogen temperature using N_2 adsorption isotherms and BET, BJH analysis methods. Samples were degassed under a vacuum condition at 200 °C overnight before measuring.

1.3.2 X-ray photoelectron spectroscopy (XPS)

X-ray photoelectron spectroscopy (XPS) was performed for the surface analysis and chemical valence state on a PHI 5000 Versaprobe II system, equipped with monochromated Al Kα radiation. The C1s peak at 284.8 eV was used as a calibration for the other peaks. Due to the overlap of Ru 3d and C 1s of the binding energies, Ru 3p spectra were recorded for the high-resolution XPS spectra. The spectra were treated with the Casa XPS software.

1.3.3 X-ray absorption fine structure analysis

Ru K-edge analysis was performed with Si (311) crystal monochromators at the BL13ssw beamlines at the Shanghai Synchrotron Radiation Facility (SSRF) (Shanghai, China). before the analysis at the beamline, the samples were pressed into thin sheets with 1 cm in diameter and sealed using Kapton tape film. The XAFS spectra were recorded at room temperature using

a 4-channel Silicon Drift Detector (SDD) Bruker 5040. Ru K-edge extended X-ray absorption fine structure (EXAFS) spectra were recorded in transmission mode. Negligible changes in the line shape and peak position of Ru K-edge X-ray absorption near-edge spectra (XANES) were observed between two scans taken for a specific sample. The XAFS spectra of these standard samples (Ru foil and RuO_2) were recorded in transmission mode. The spectra were processed and analyzed by the software codes Athena and Artemis.

1.4. Catalytic reactions and product analysis

The direct hydrodeoxygenation of lignin was performed in a 160 ml stainless Hastelloy autoclave (Parr reactor). In a typical run, feedstock (lignin 0.1 g), catalyst (0.1 g), and dodecane (20 ml) were loaded into the autoclave and sealed. Herein, both the stirring bar and the sensing tip of the thermal couple are fully immersed in the solvent. After air purging with N_2 three times, the reactor was heated to the designed temperature (250 °C) under vigorous stirring. Once the target temperature was maintained, the reactor was pressurized to 5 MPa with H_2. Once the reaction finished, the autoclave was cooled quickly to room temperature in an ice water bath. The products in the liquid phase were qualitatively analyzed by GC-MS and quantitatively analyzed by a GC-flame ionization detector equipped with a VF-Xms Column. The vaporization temperature was 250 °C, and the oven temperature was from 40 °C to 270 °C at a rate of 10 °C/min.

The corresponding response factors were determined by analyzing mixtures of pure, commercially obtained liquid hydrocarbons and an internal standard with precisely known masses. The corresponding response factors of oxygenated species were evaluated by the effective carbon numbers method. The yields of liquid alkanes were determined and calculated by adding decalin as the internal standard after the reaction. The mass yield of the product (C_i) was calculated by the following equation:

$$\text{mass yield } (C_i,\%) = \frac{\text{mass of } C_i}{\text{mass of feedstock input}} \times 100\%$$

$$= \frac{\text{Area } (C_i)}{\text{Area of internal standard}} \times f \times \frac{\text{mass of internal standard}}{\text{mass of feedstock input}} \times 100\%$$

where: f is the relative response factor.

The carbon yield was calculated by the equation:

$$\text{carbon yield } (C_i,\%) = \frac{\text{mass of carbon in } C_i}{\text{mass of carbon in initial feedstock}} \times 100\%$$

1.5 Near ambient pressure XPS (NAP-XPS) measurements

Near ambient pressure X-ray photoelectron spectroscopy (NAP-XPS) was conducted on a Specs NAP-XPS instrument at the Vacuum Interconnected Nanotech Workstation (Nano-X), where XPS (ESCALAB 250Xi) was connected to a glovebox (Vigor) via ultra-high vacuum tubes to prevent air contamination. The catalysts were heated in H_2 to the target temperature by a temperature-controlled laser heating device, and the photon source is the monochromatic X-ray source of Al Ka (1486.6 eV). The catalysts were reduced at 300 °C in a H_2 pressure of 0.2 mbar.

1.6 Density functional theory (DFT) calculation

Density functional theory (DFT) calculations were conducted using the Perdew-Burke-Ernzerhof (PBE) functional under the generalized gradient approximation (GGA),[2] implemented in the Vienna ab initio simulation package (VASP, version 6.4.2).[3] The interaction between core and valence electrons was described via the projector-augmented wave (PAW) method.[4] A plane-wave basis set with a 450 eV energy cutoff was employed for expanding the valence electronic states. Transition states (TS) were located using a constrained optimization scheme,[5] and structural optimizations were terminated when the maximum force on any relaxed atom was below 0.05 eV/Å. Due to the large supercell size (17 × 17 × 30 Å), a

1 × 1 × 1 k-point mesh was sufficient. This DFT setup, validated by earlier studies, provides reliable accuracy.[6-8] The molecular dynamics (MD) simulation for Ru/CNF-700 was conducted over a time span from 0 ps to 7 ps at a temperature of 573 K. Adsorption energies were determined using $E_{ads}(X) = E_{X/surf} – E_{surf} – E_X$, where E_X, E_{surf}, and $E_{X/surf}$ are the total energies of the gas-phase adsorbate, the surface, and the surface with the adsorbate, respectively.[9,10] Due to the large supercell size (17 × 17 × 30 Å), a 1 × 1 × 1 k-point mesh was sufficient. 3 graphene layers were used to build the model. To mimic the real catalysts, after the structure optimization, Ru_{18} clusters were used. To distinguish CNF surfaces with different oxygen-containing group coverages, we constructed different representative models. First, for the Ru/CNF-700 system, in which the oxygen-containing group coverage was reduced due to calcination at 700 °C, we introduced 5 OH groups onto the model surface, designating it as OH-deficient. Second, for the Ru/CNF system dried at 100 °C, we introduced 16 OH groups onto the model surface, denoted as OH-rich. Third, following OH–OH coupling on the Ru/CNF surface, O groups were formed, and this model was labeled as O-rich. This DFT setup, validated by earlier studies, provides reliable accuracy.[6-8]

Density functional theory (DFT) calculations are employed to gain fundamental insights into the role of surface oxygen chemistry in hydrogen activation and C-O bond cleavage during lignin hydrodeoxygenation (HDO). Following well-established modeling approaches in biomass conversion, phenol is used as a representative model compound, and idealized Ru_{18} cluster models with varying oxygen coverages are constructed to explore qualitative trends. While these simplifications do not capture the full complexity of the real catalytic system, such as solvent effects, high-pressure hydrogen, or competitive adsorption, they allow us to extract key mechanistic principles and site requirements that help rationalize experimental observations and inform catalyst design.

Supplementary figures and Tables

Table S1. Physical properties of the Ru catalysts.

	Surface area (m^2/g)	Pore volume (cm^3/g)	Pore size (Å)
Ru/AC	811.2	0.77	94.4
Ru/Al_2O_3	106.7	0.72	199.0
Ru/CNF	153.6	0.40	97.8
Ru/CNF-700	152.7	0.39	97.1
Ru/CNF-1000	152.9	0.39	97.2

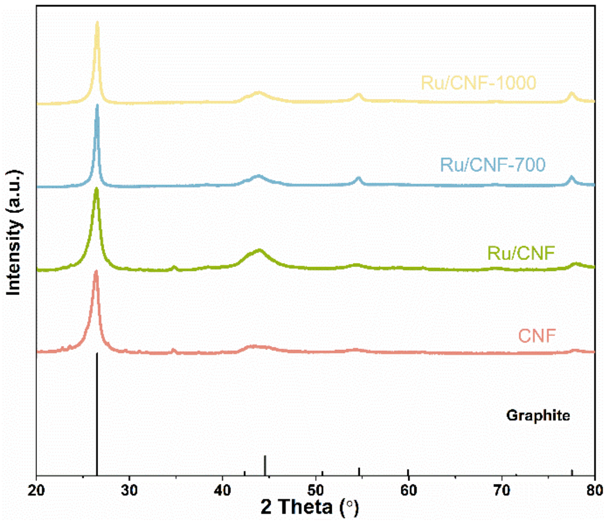

Figure S1. XRD patterns of CNF and Ru/CNF catalysts

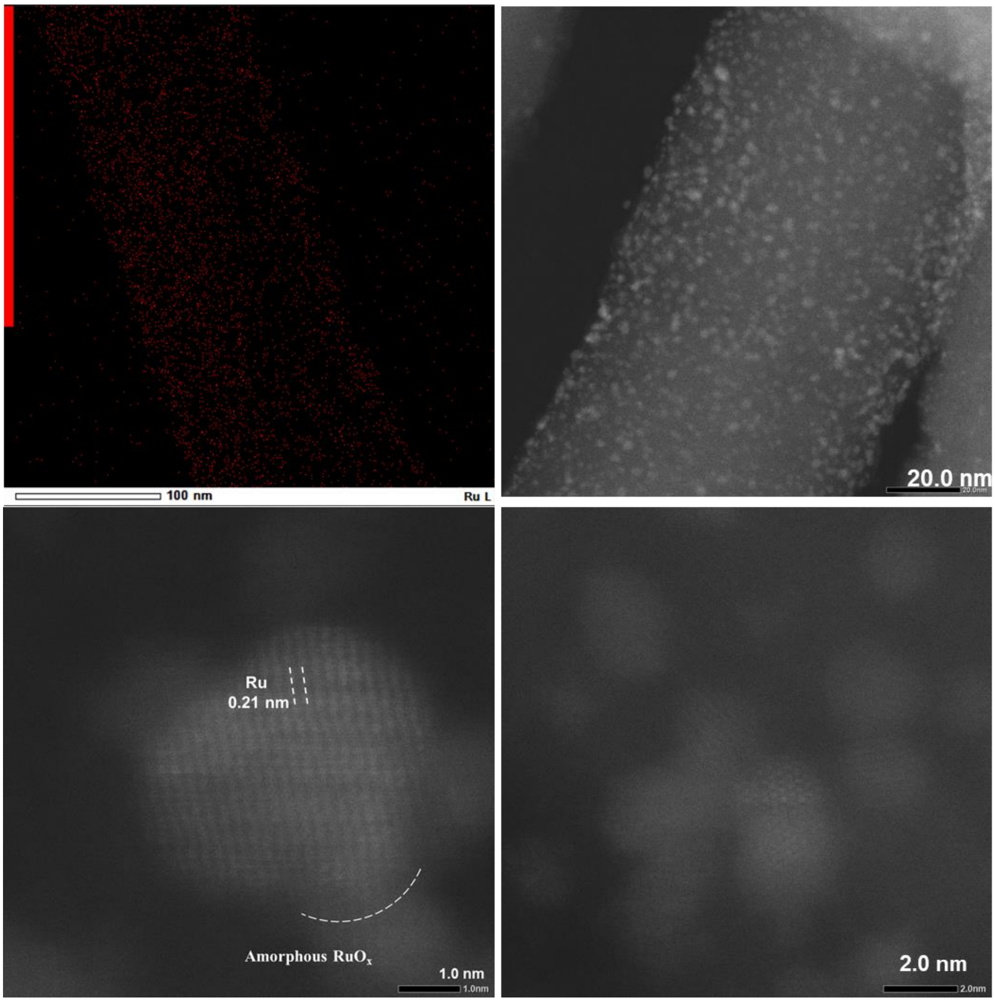

Figure S2. STEM-EDX elemental mapping of Ru/CNF catalyst.

Table S2. Ru K-edge EXAFS fitting parameters of Ru/CNF. (Notes: N, coordination number; R, the internal atomic distance; ΔE_0, the edge-energy shift; σ^2, Debye-Waller factor.)

	Path	N	R(Å)	ΔE_0 (eV)	σ^2 (Å^2)	R factor
Ru/CNF	Ru−Ru	2.7± 1.4	2.70± 0.02	-2.2± 3.4	0.007±0.003	0.01
	Ru−O1	2.6± 0.8	1.89± 0.20	-4.9± 4.1	0.005± 0.002	
	Ru−O2	3.2± 4.9	2.03± 0.06	-4.9± 4.1	-0.002±0.001	

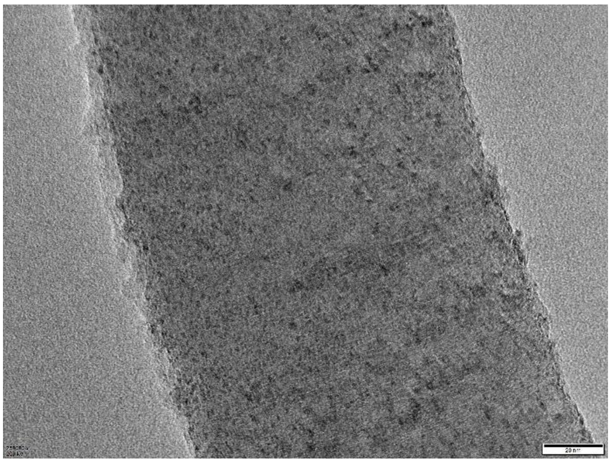

Figure S3. HR-TEM images of Ru/CNF-700.

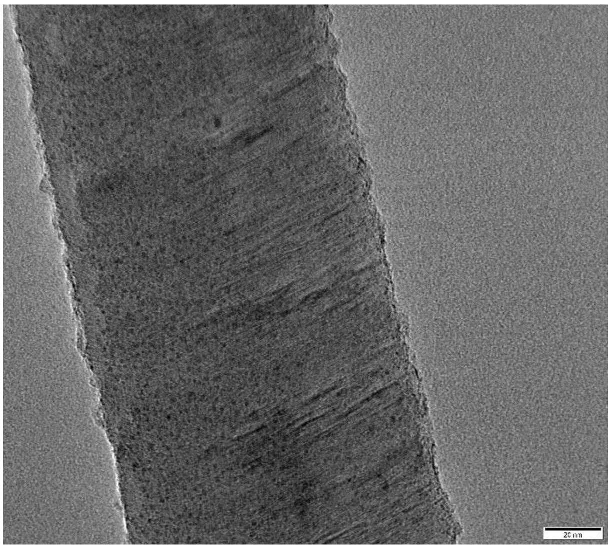

Figure S4. HR-TEM images of Ru/CNF-1000.

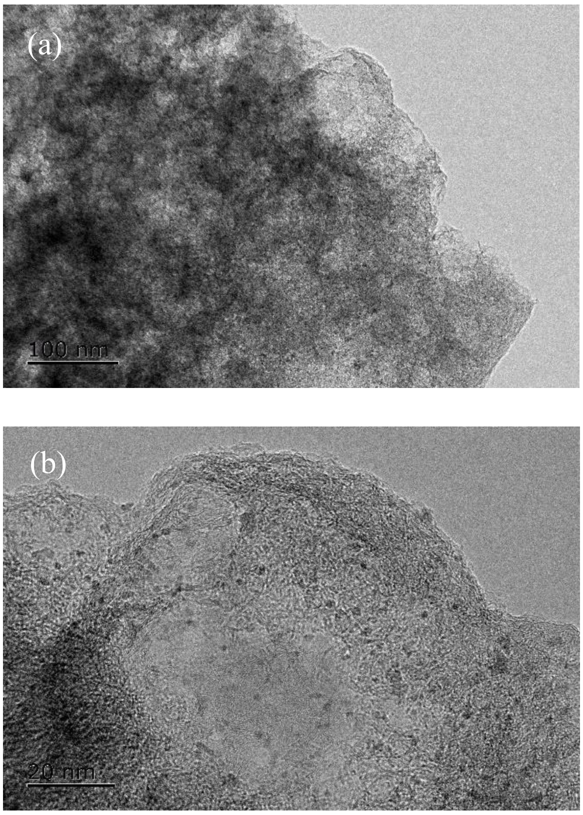

Figure S5. (a, b) HR-TEM images of Ru/AC catalyst.

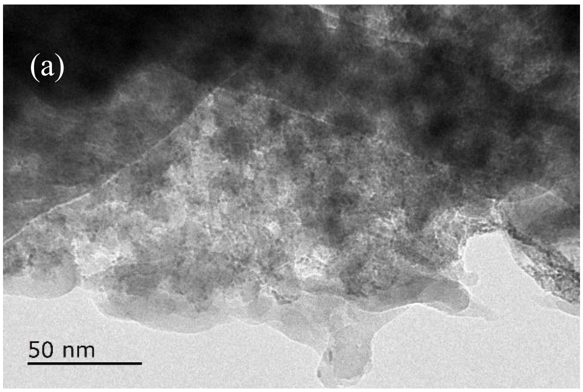

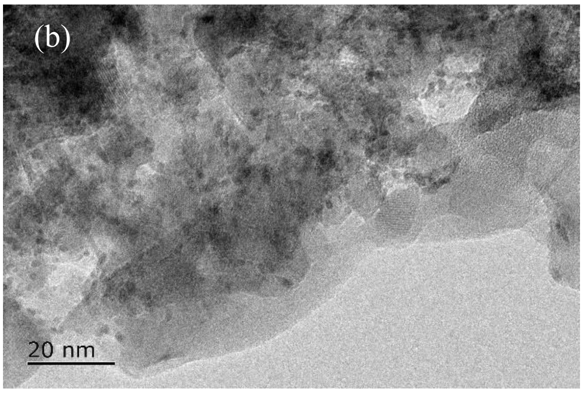

Figure S6. (a, b) HR-TEM images of Ru/Al_2O_3 catalyst.

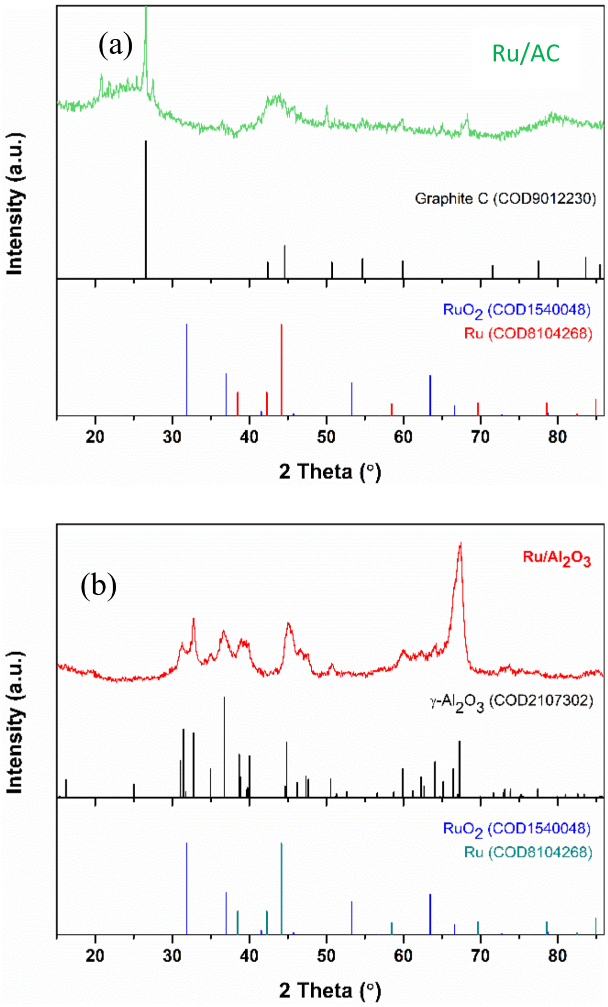

Figure S7. XRD patterns of the fresh (a) Ru/AC, and (b) Ru/γ-Al_2O_3 catalysts with Ru, RuO_2, and carbon standards.

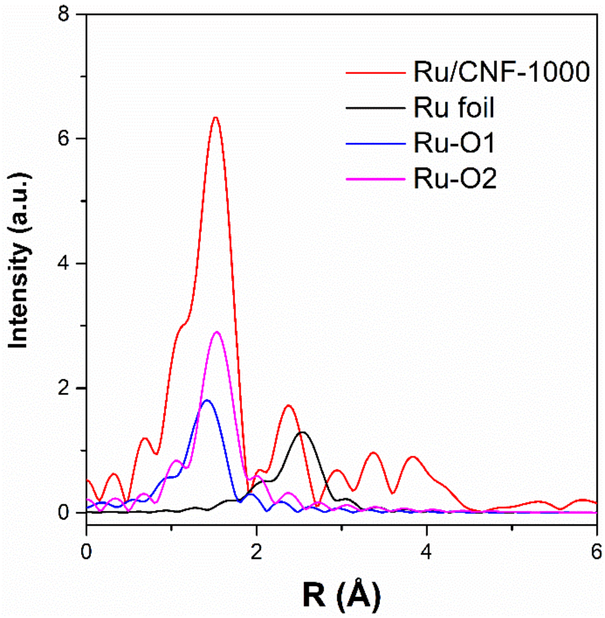

Figure S8. FT-EXAFS spectra of Ru K-edge of Ru/CNF-1000 and Ru foil and RuO_2.

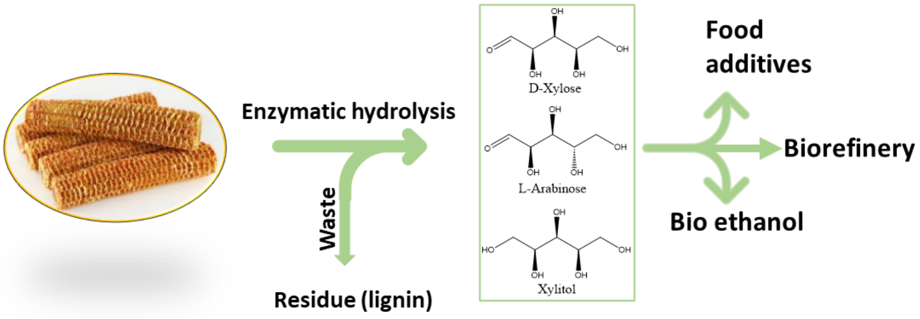

Figure S9. The illustration on the process of sugar production from corncob.

Table S3. Summary of the converting lignin to liquid hydrocarbons from lignin and woody biomass. (TW: this work)

<table>
<tr><th>Catalyst</th><th>T (°C)</th><th>P (MPa)</th><th>Feedstock</th><th>Main products</th><th>Mass yield</th><th>Main products distribution</th><th>Ref.</th></tr>
<tr><td rowspan="3">Ir-ReO_x/SiO_2</td><td rowspan="3">260</td><td rowspan="3">4</td><td>Organosolv lignin</td><td rowspan="3">Cycloalkanes</td><td>19.3%</td><td>Cyclohexane: 3.2%
Methyl cyclohexane: 6.7%
Ethyl cyclohexane: 2.2%
Propyl cyclohexane: 7.2%</td><td rowspan="3">11</td></tr>
<tr><td>Enzymolysis lignin</td><td>9.8%</td><td>Cyclohexane: 3.6%
Methyl cyclohexane: 0.7%
Ethyl cyclohexane: 3.6%
Propyl cyclohexane: 1.9%</td></tr>
<tr><td>Alkaline lignin</td><td>6.6%</td><td>Cyclohexane: 3.1%
Methyl cyclohexane: 0.5%
Ethyl cyclohexane: 2.2%
Propyl cyclohexane: 0.8%</td></tr>
<tr><td>Pt/$NbOPO_4$</td><td>190</td><td>5</td><td>Birchwood</td><td>Pentanes, hexanes, alkylcyclohexanes</td><td>28.1%</td><td>Pentanes: 10.2%
Hexanes: 13.1%
Alkylcyclohexanes: 4.8%</td><td>12</td></tr>
<tr><td rowspan="2">Pd/m-MoO_3-P_2O_5/SiO_2</td><td>180</td><td>1</td><td rowspan="2">Bio-oil</td><td rowspan="2">Pentane, hexane, methylcyclopentane, cycohexanes</td><td>9.4%</td><td>Pentane: 0.9%
Hexane: 1.2%
Methylcyclopentane: 0.9%
Cyclohexanes: 5.6%</td><td rowspan="2">13</td></tr>
<tr><td>250</td><td>1</td><td>29.6%</td><td>Pentane: 7.4%
Hexane: 5.1%
Methylcyclopentane: 3.7%
Cyclohexanes: 13.4%</td></tr>
<tr><td>Ru/Nb_2O_5</td><td>250</td><td>0.7</td><td>Birch lignin</td><td>Arenes, cyclohexanes</td><td>29.7%</td><td>Toluene: 2.8%
Ethyl benzene: 9.1%
Propyl benzene: 8.5%
Methyl cyclohexane: 0.6%
Ethyl cyclohexane: 4.4%
Propyl cyclohexane: 3.6%</td><td>14</td></tr>
<tr><td>Ni/SiO_2-Al_2O_3</td><td>300</td><td>6</td><td>Lignin</td><td>Alkanes</td><td>42%</td><td>Alkanes: 42%</td><td>15</td></tr>
<tr><td>NiAl alloy</td><td>220</td><td>2</td><td>Poplar wood sawdust</td><td>Aromatic monomers</td><td>18.9%</td><td>Aromatic monomer: 18.9%</td><td>16</td></tr>
<tr><td>Ru/Nb2O5-SiO2</td><td>230</td><td>-</td><td>Birch lignin</td><td>Arenes</td><td>19.8%</td><td>Toluene: 1.8%
Ethyl benzene: 8.0%
Propyl benzene: 5.0%
arene dimers: 3.8%
Cycloalkanes: 1.2%</td><td>17</td></tr>
<tr><td>Ni-Cu/H-Beta zeolite</td><td>330</td><td>-</td><td>Kraft lignin</td><td>Cycloalkanes</td><td>40.39%</td><td>Cycloalkanes: 40.39%</td><td>18</td></tr>
<tr><td>Ru/CNF</td><td>250</td><td>5 (at 250 °C)</td><td>Corncob</td><td>Cycloalkanes</td><td>49.1%</td><td>Cycloalkanes (C_6: 26.5%, C_7: 12.6%, C_8: 8.0%, C_9: 2.0%)</td><td>TW</td></tr>
</table>

Note: the pressures were reported as the initial pressure at room temperature in the literature.

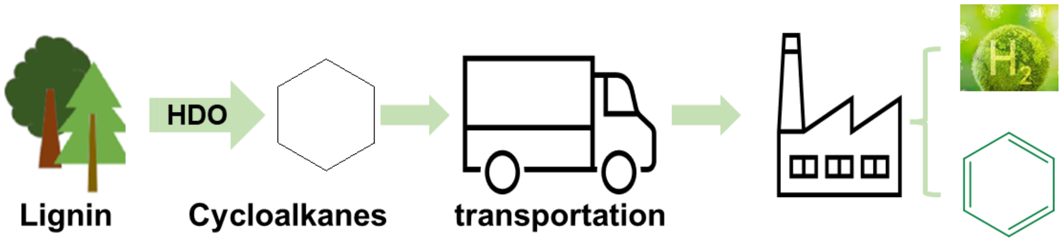

Figure S10. The concept of cycloalkane as the benzene and H_2 carrier in the HDO process.

This research not only provides a significant advancement in the one-pot HDO of lignin to hydrocarbons but also offers a promising approach for integrated hydrogen storage and the production of green aromatics through the selective conversion of lignin with green hydrogen to cycloalkanes, as shown in Figure S10. The use of cycloalkanes as a hydrogen carrier further highlights the potential of this process in facilitating the transportation and on-site release of hydrogen, underscoring the environmental and commercial viability of converting lignin, a byproduct of the biorefinery industry, into valuable products using Ru/CNF catalysts.

Table S4. Summary of product yields of direct HDO of lignin over different catalysts.

Catalyst	Mass yield (wt%)								Total (%)
	Cycloalkanes				Aromatics				
	C_6	C_7	C_8	C_9	C_6	C_7	C_8	C_9	
Ru/CNF	26.5	12.6	8.0	2.0	-	-	-	-	49.1
Ru/CNF-700	7.3	2.8	5.2	-	-	-	-	0.8	16.1
Ru/CNF-1000	0.8	3.0	4.4	1.8	-	-	-	1.3	11.3
Ru/AC	0.7	2.4	1.6	0.8	3.8	2.5	1.9	0.5	14.2
Ru/Al_2O_3	0.3	1.3	0.3	-	3.0	2.2	1.8	0.7	9.6

Reaction conditions: 0.1 g lignin, W_{cat}=0.1 g, 20 ml dodecane, 8 h, 5 MPa H_2 at 250 °C.

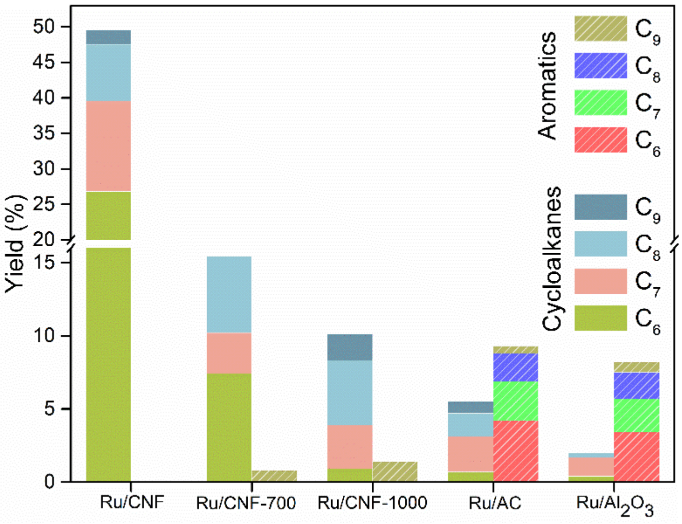

Figure S11. Catalytic performance of HDO of lignin. Comparison of carbon numbers of different catalysts at identical reaction conditions. Reaction conditions: 0.1 g lignin, W_{cat}=0.1 g, 20 ml dodecane, 5 MPa H_2 at 250 °C, 8 h.

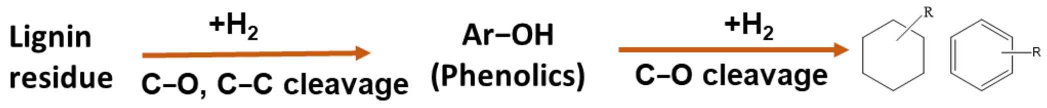

Figure S12. Steps of converting lignin to liquid hydrocarbons.

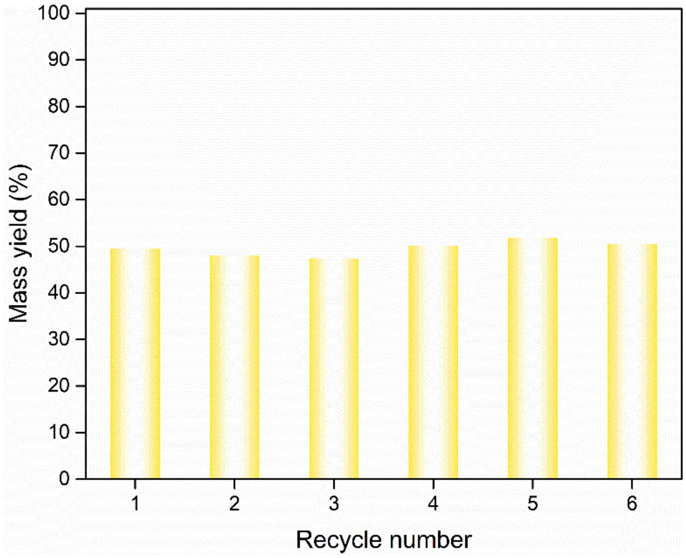

Figure S13. Stability test of Ru/CNF in batch reactions. Reaction conditions: 0.1 g lignin, Wcat=0.1 g, 20 ml dodecane, 5 MPa H_2 at 250 °C, 8 h.

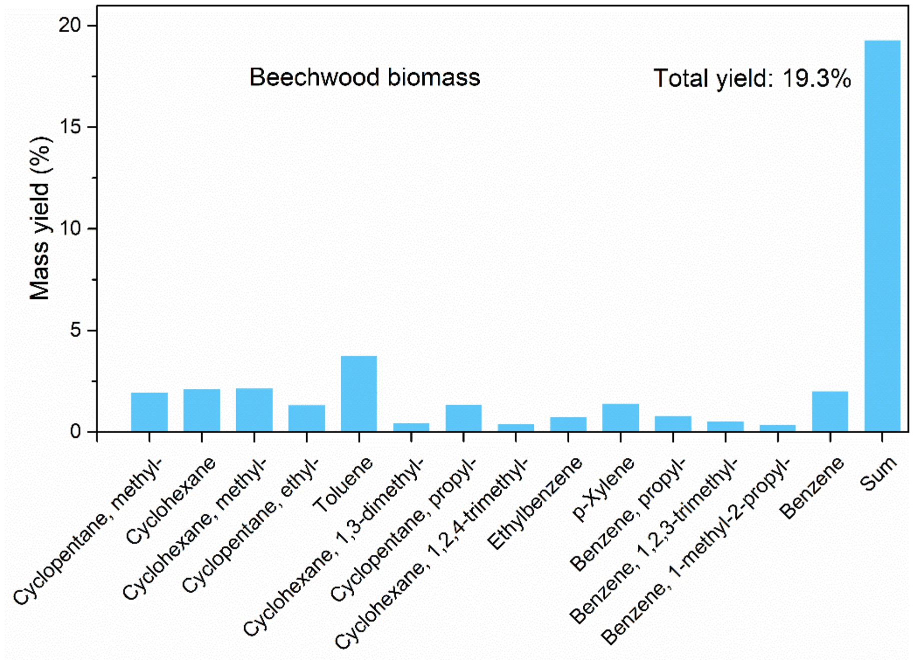

Figure S14. Catalytic performance of HDO of beechwood biomass of Ru/CNF catalyst. Reaction conditions: 0.1 g biomass, 0.1 g catalyst, 20 ml dodecane, 8 hours reaction, 5 MPa H_2 at 250 °C.

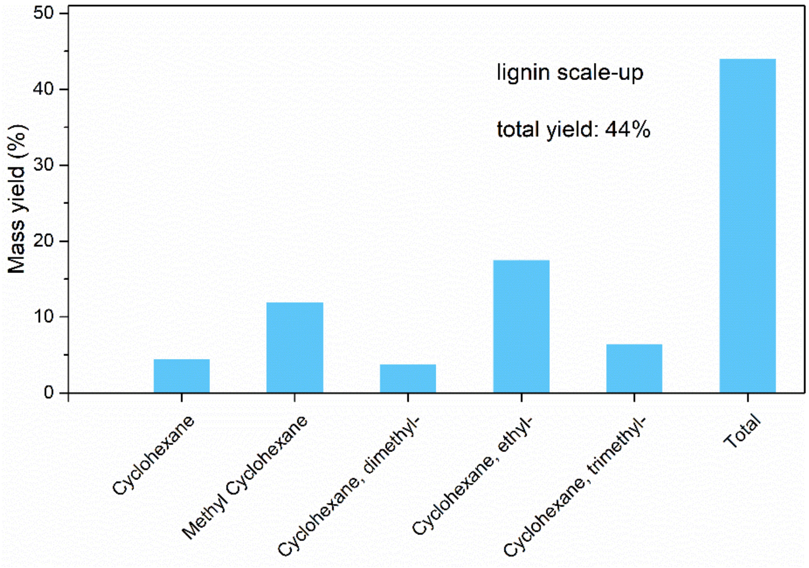

Figure S15. Catalytic performance of HDO of lignin over Ru/CNF catalyst. Reaction conditions: 0.5 g biomass, 0.5 g catalyst, 20 ml dodecane, 8 hours reaction, 10 MPa H_2 at 250 °C.

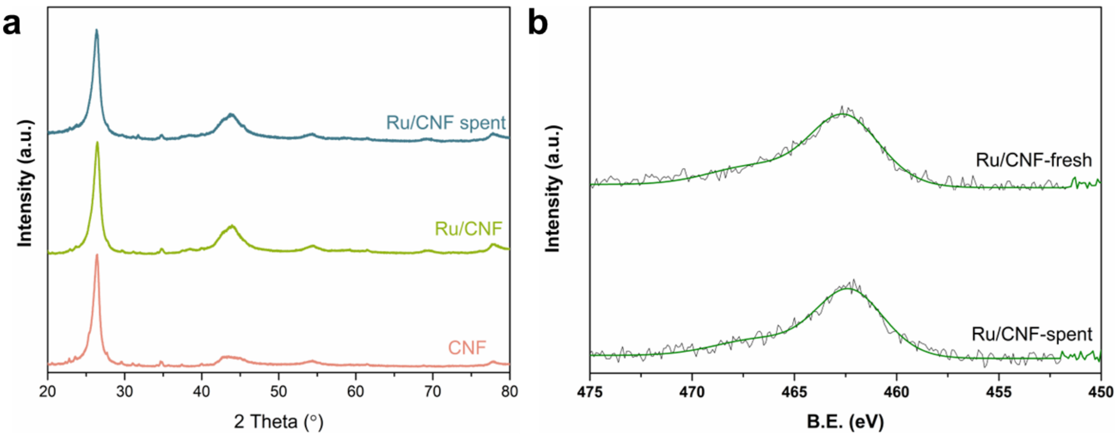

Figure S16. Stability test of Ru/CNF in batch reactions. (a) XRD patterns of the fresh and spent Ru/CNF catalysts. (b) XPS spectra of Ru 3p of the Ru/CNF fresh (top) and spent (bottom) catalysts. Reaction conditions: 0.1 g lignin, W_{cat}=0.1 g, 20 ml dodecane, 8 h, 5 MPa H_2 at 250 °C.

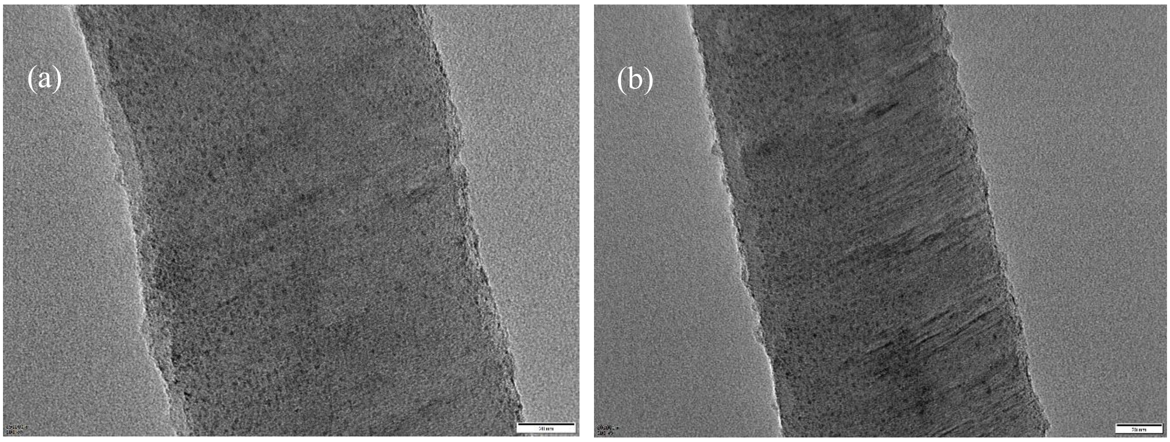

Figure S17. HR-TEM images of Ru/CNF catalyst. (a) fresh catalyst, (b) used catalyst.

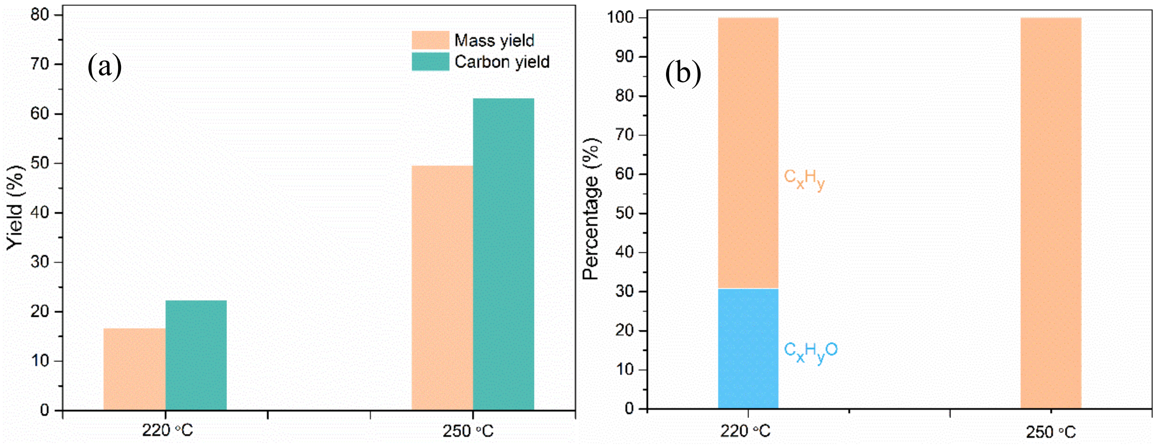

Figure S18. Catalytic performance of HDO of lignin at different temperatures of Ru/CNF catalyst. Reaction conditions: 0.1 g lignin, 0.1 g catalyst, 20 ml dodecane, 8 hours reaction, 5 MPa H_2 at 250 °C or 220 °C.

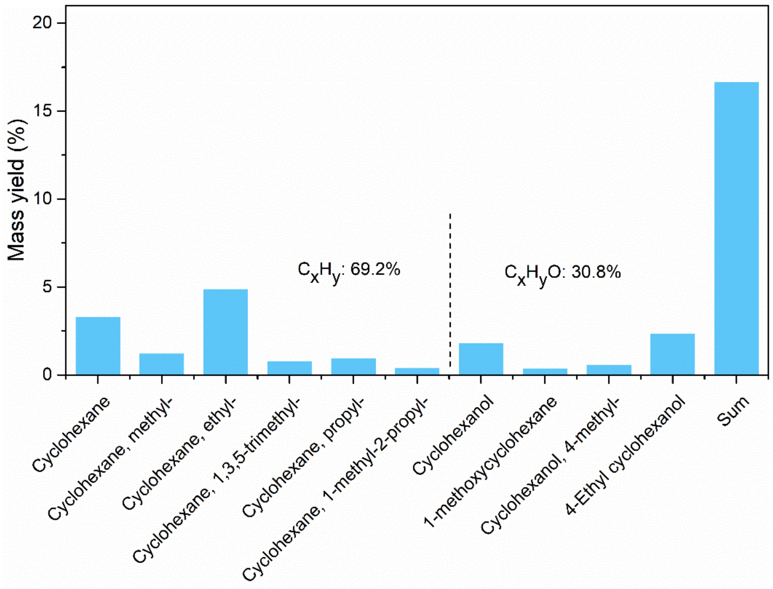

Figure S19. Catalytic performance of HDO of lignin on product distribution of Ru/CNF catalyst. Reaction conditions: 0.1 g lignin, 0.1 g catalyst, 20 ml dodecane, 8 hours reaction, 5 MPa H_2 at 220 °C.

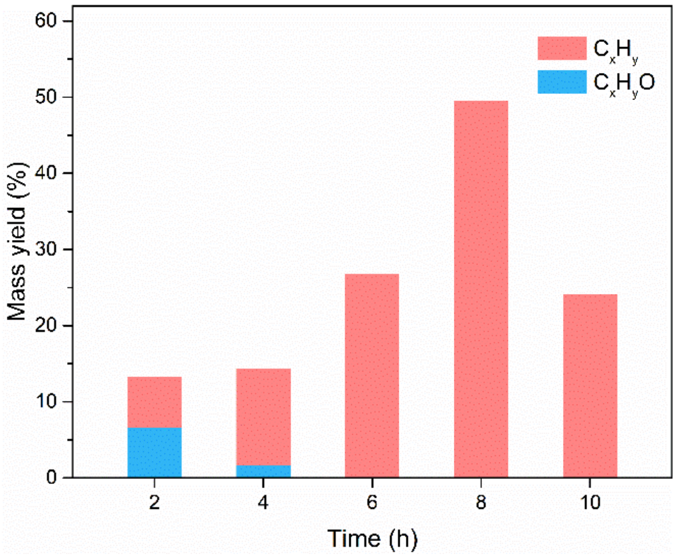

Figure S20. Catalytic performance of HDO of lignin at different reaction times of Ru/CNF catalyst. Reaction conditions: 0.1 g lignin, 0.1 g catalyst, 20 ml dodecane, 5 MPa H_2 at 250 °C.

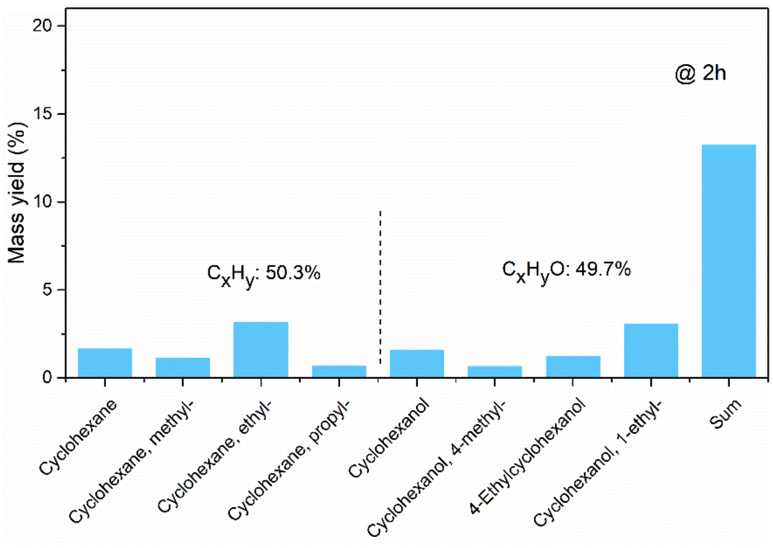

Figure S21. Catalytic performance of HDO of lignin of Ru/CNF catalyst. Reaction conditions: 0.1 g lignin, 0.1 g catalyst, 20 ml dodecane, 5 MPa H_2 at 250 °C, 2 hours.

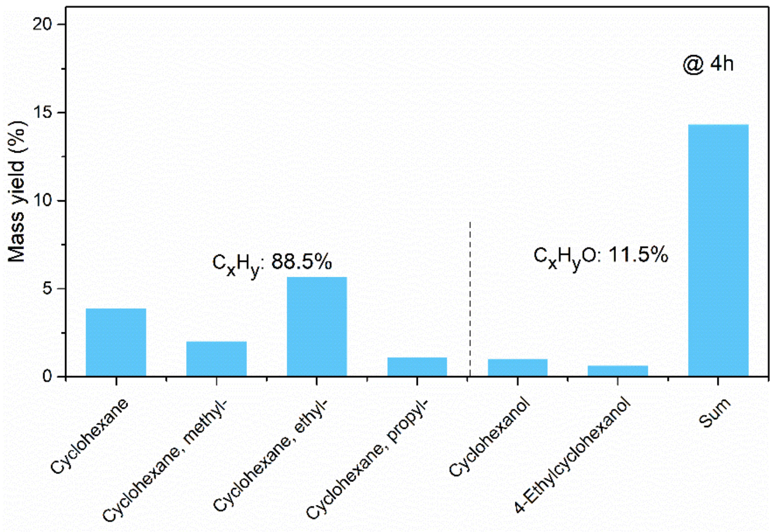

Figure S22. Catalytic performance of HDO of lignin of Ru/CNF catalyst. Reaction conditions: 0.1 g lignin, 0.1 g catalyst, 20 ml dodecane, 5 MPa H_2 at 250 °C, 4 hours.

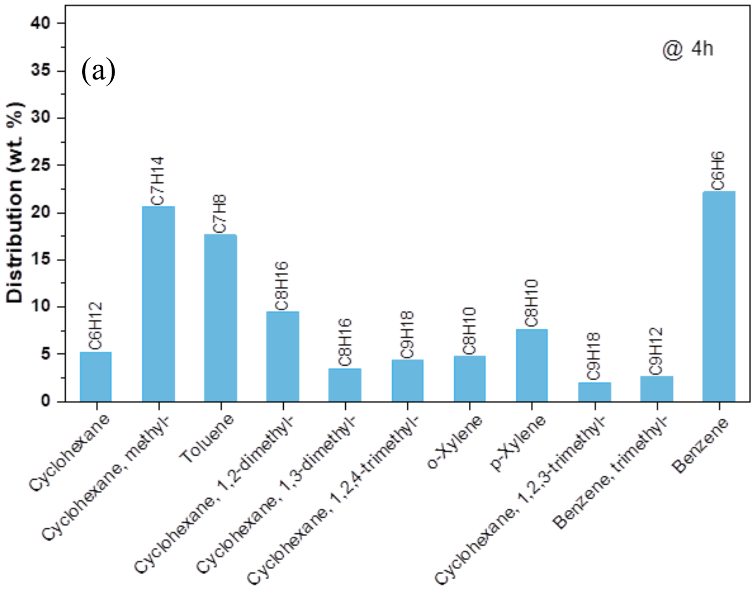

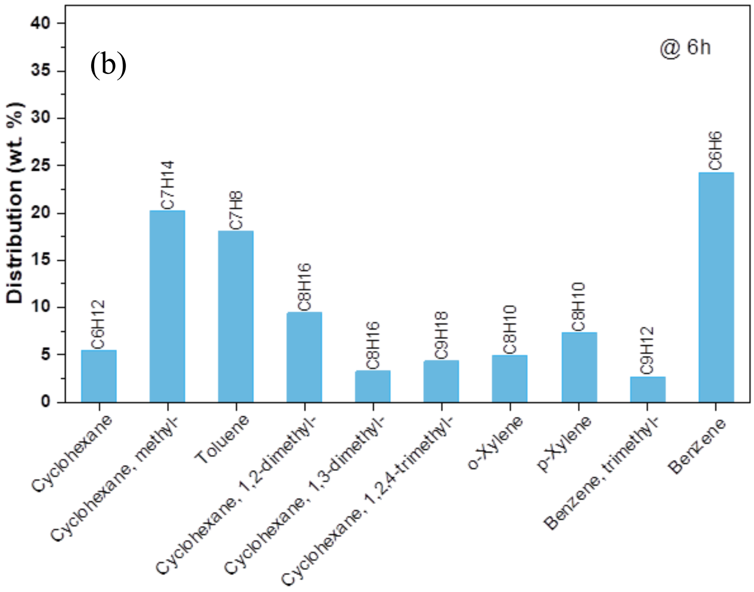

Figure S23. Product distributions of direct HDO of lignin over Ru/AC catalyst. Reaction conditions: 0.1 g lignin, 0.1 g Ru/AC, 20 ml dodecane, 5 MPa H_2 at 250 °C, (a) 4 hours, and (b) 6 hours reaction.

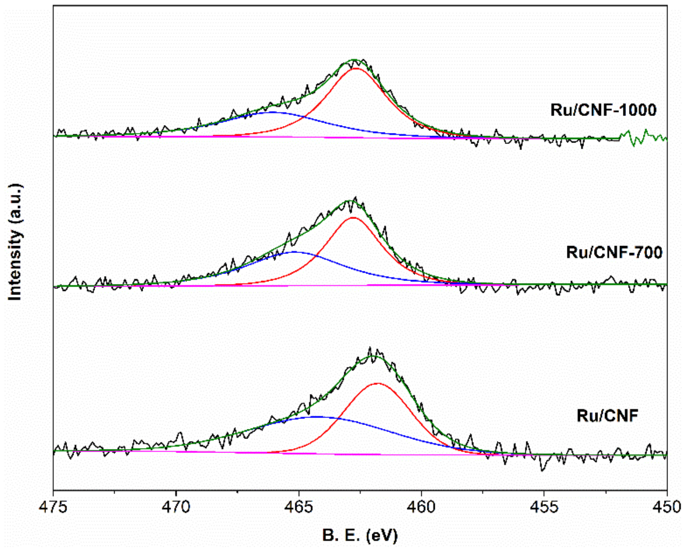

Figure S24. High resolution Ru 3p spectra of the CNF catalysts.

Table S5. Summary of the Ru 3p XPS spectra of the Ru/CNF catalysts.

	Ru^0	Ru^{n+}
Ru/CNF	47.2%	52.8%
Ru/CNF-700	54.2%	45.8%
Ru/CNF-1000	62%	38%

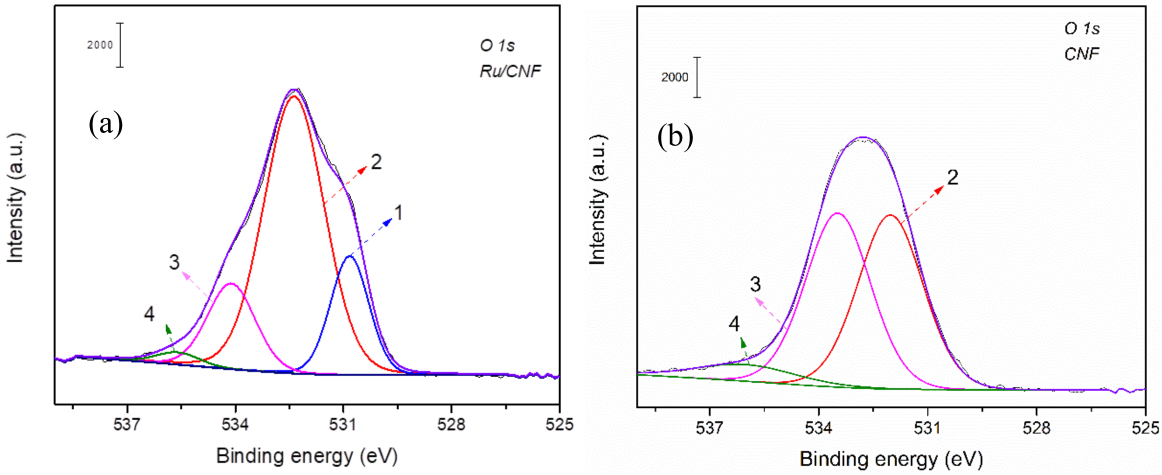

Figure S25. XPS high-resolution O 1s spectra of (a) Ru/CNF, (b) CNF.

Table S6. Summary of the O 1s spectra of CNF and Ru/CNF catalysts.

	Peak 1 (Ru-O)	Peak 2 (hydroxyl)	Peak 3 (ester)	Peak 4 (carboxyl)
CNF	-	532.0 eV<br>46.53%	533.5 eV<br>46.52%	535.4 eV<br>6.95%
Ru/CNF	530.3 eV<br>17.6%	531.9 eV<br>64.63%	533.66 eV<br>15.76%	535.15 eV<br>2.02%

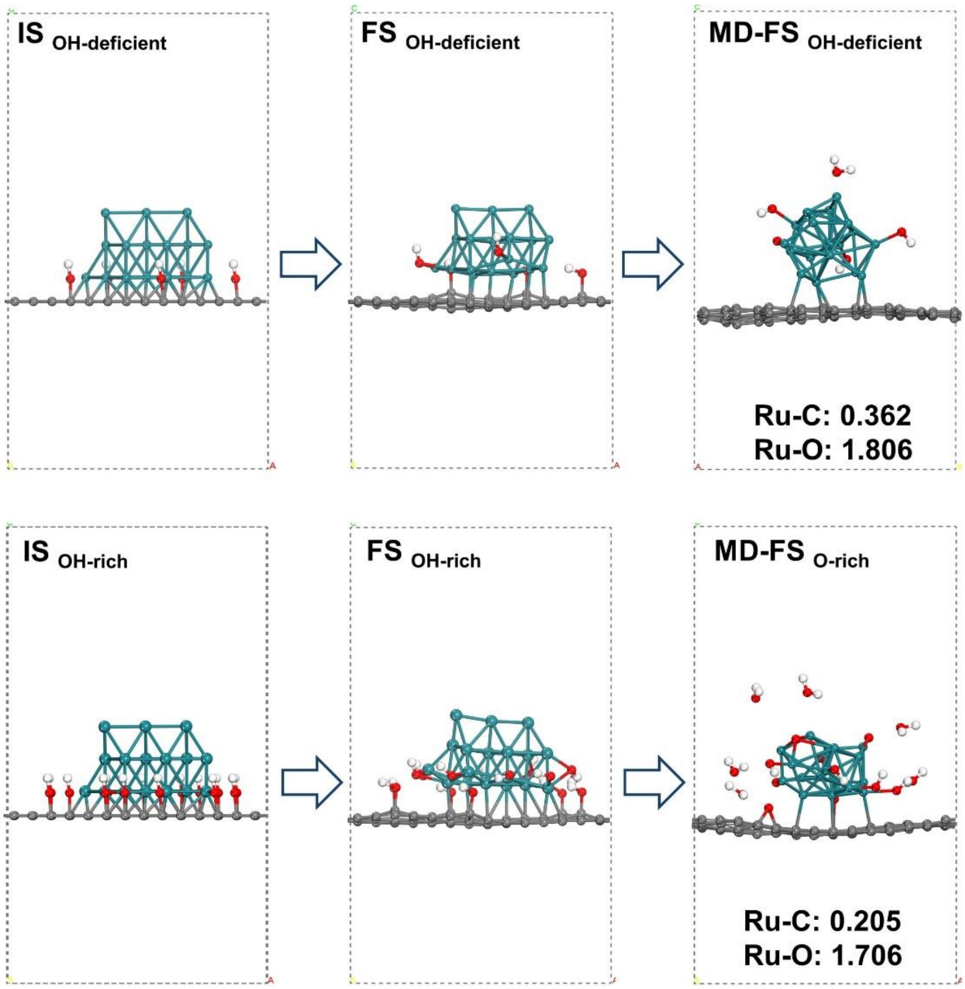

Figure S26. Side views of the thermodynamically optimized initial state (IS) and final state (FS), as well as the molecular dynamics evolved final states (MD-FS) on OH-rich and OH-deficient models. The Ru-C and Ru-O bond energy (-ICOHP) at the MD-FS state is indicated in black.

Table S7. Summary of the transtion state and adsorption configurations involved in the HDO process.

	$Ru_{18}(OH)_5$	$Ru_{18}(OH)_{16}$	$Ru_{18}(OH)_8O_4$
H_2 adsorption	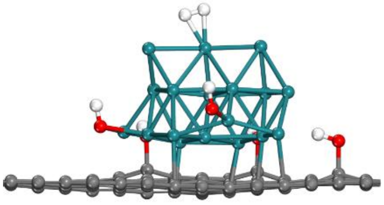<br>-0.25 eV	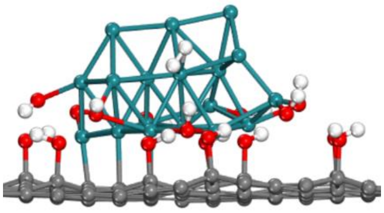<br>-0.67 eV	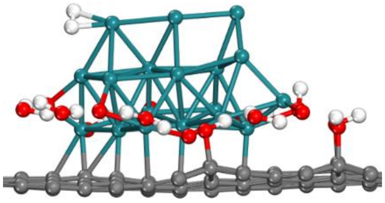<br>-1.01 eV
H_2 dissociation (Transition state)	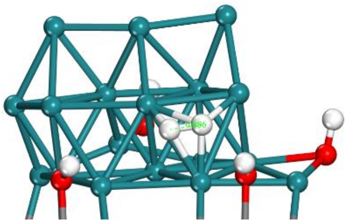<br>0.23 eV	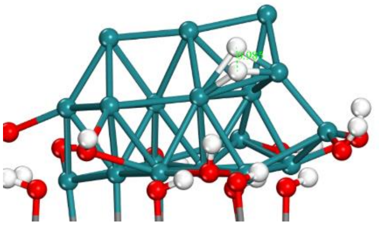<br>0.15 eV	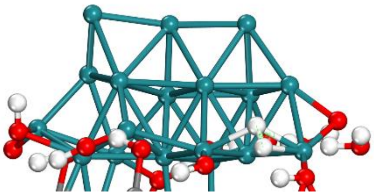<br>0.09 eV
Phenol adsorption	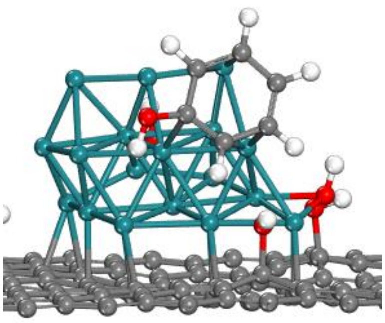<br>-1.85 eV	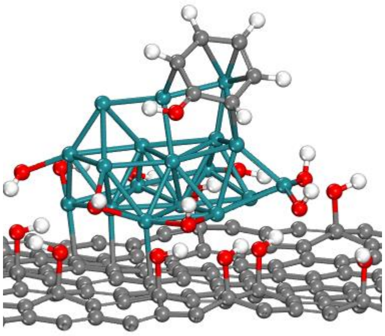<br>-1.85 eV	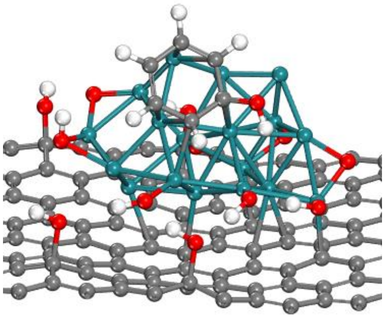<br>-1.92 eV
Phenol dissociation (transiton state)	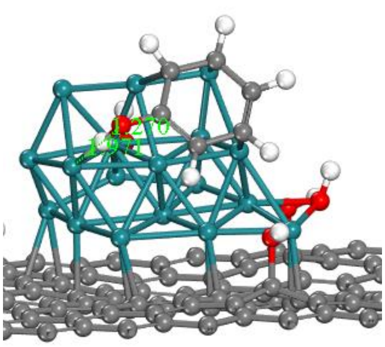<br>0.59 eV	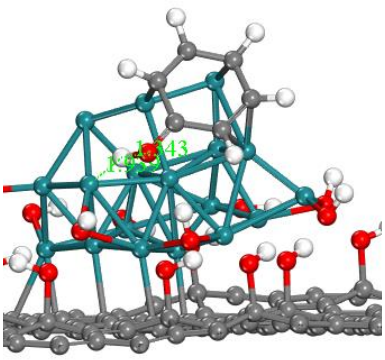<br>0.28 eV	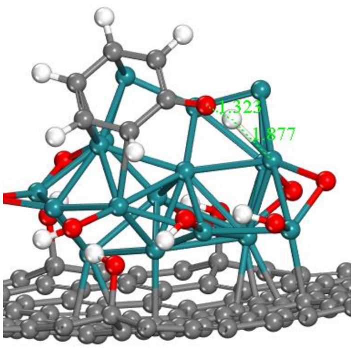<br>0.17 eV

Phenol addition (transtion state)	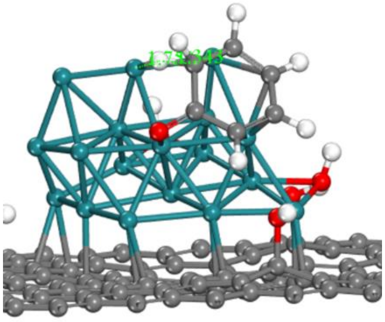<br>1.54 eV	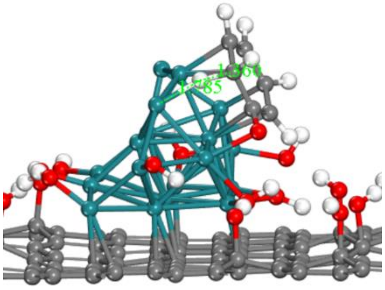<br>0.65 eV	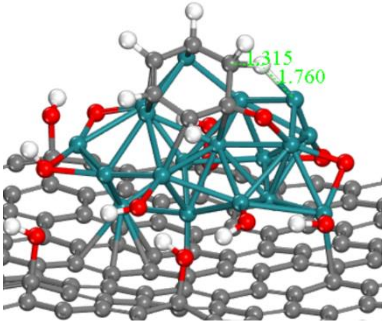<br>0.50 eV
C-O cleavage of cyclohexanol (transtion state)	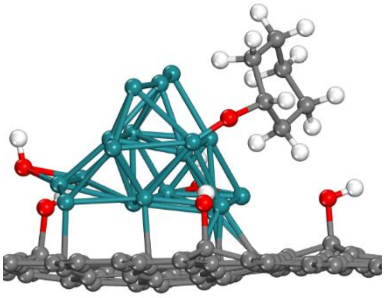<br>0.69 eV	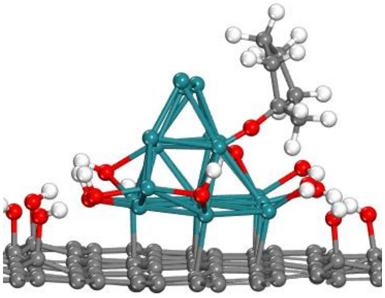<br>0.40 eV	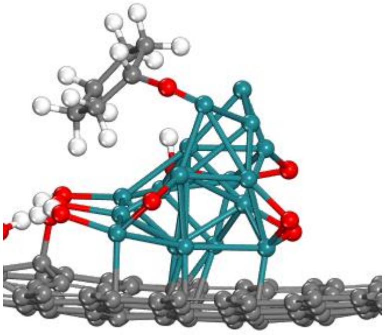<br>0.17 eV
C-O cleavage of phenol (transtion state)	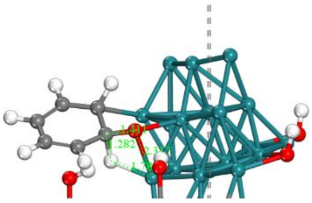<br>1.04 eV	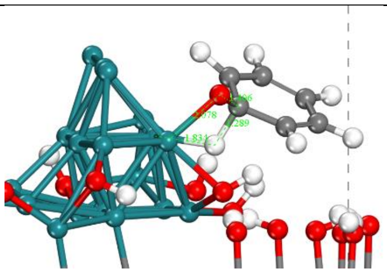<br>0.63 eV	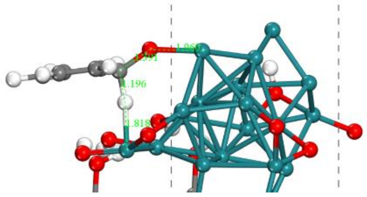<br>0.60 eV
Benzene addition (transtion state)	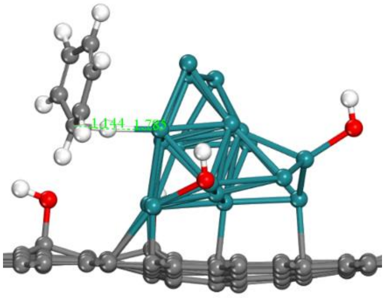<br>2.12 eV	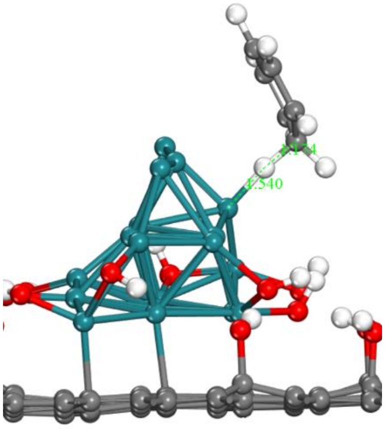<br>0.99 eV	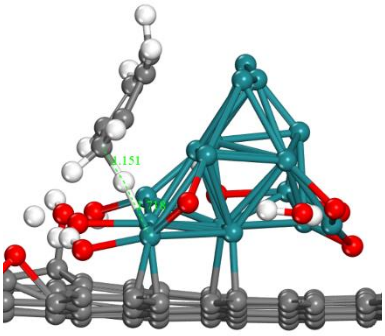<br>0.75 eV

Table S8. Bader charge analysis for the transition state of H_2 dissociation of the Ru/CNF catalysts.

	$Ru_{18}(OH)_{16}$	$Ru_{18}(OH)_8O_4$
Initial state	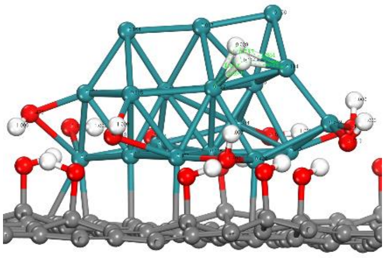	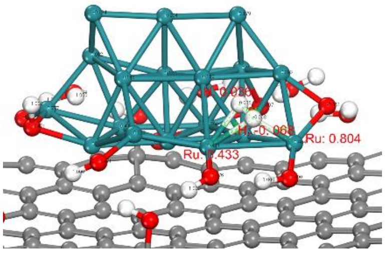
Transition state	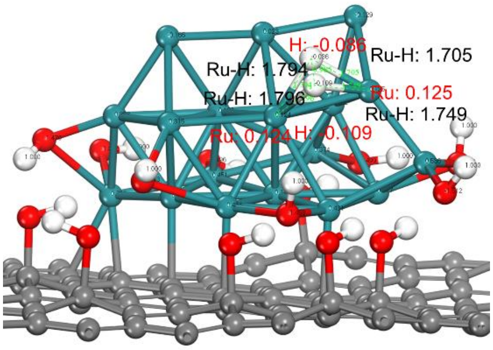 	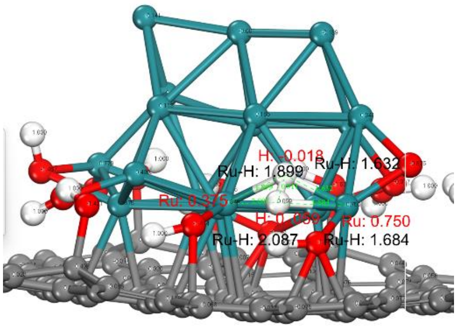
Final state	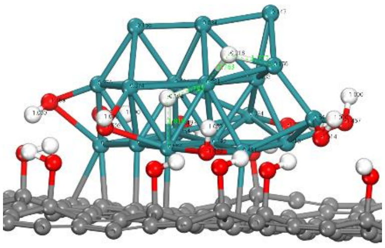	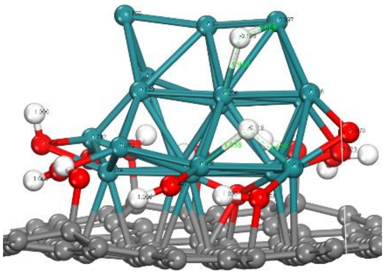

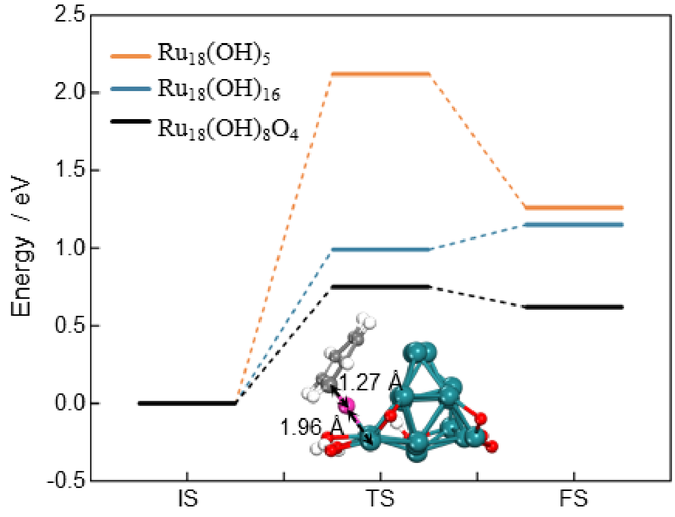

Figure S27**.** Energy profiles for the hydrogenation of benzene intermediates, with insets showing the transition state structures. Bond lengths at the transition state are indicated in black.

Supplementary note 1: phenol adsorption and dissociation

Phenol adsorbs on Ru surfaces primarily through its aromatic π system. As shown in Figure 5a and Table S6, phenol binds strongly on all three Ru/CNF models, with adsorption energies of -1.85 eV for both $Ru_{18}(OH)_5$ and $Ru_{18}(OH)_{16}$, and a slightly stronger adsorption of -1.92 eV on $Ru_{18}(OH)_8O_4$. This small variation indicates that phenol adsorption is dominated by π–metal interactions, but the O-rich surface provides a somewhat stronger binding environment due to enhanced electron depletion of surface Ru atoms. In contrast to the relatively similar adsorption energies, the dissociation of the phenolic O-H bond depends strongly on the polarity and charge asymmetry of the surface-active sites.

O-H activation occurs through its interaction with a polarized $Ru^{\delta+}$ center, forming a $C_6H_5O\cdots H\cdot\cdot Ru^{\delta+}–O^{\delta-}(OH^{\delta+})$ transition-state configuration that reflects partial heterolytic splitting of the O-H bond. The ease of forming this transition state varies dramatically across the three surfaces:

On the O-rich surface ($Ru_{18}(OH)_8O_4$), phenolic O–H dissociation is the most favorable, with a very low barrier of 0.17 eV. This arises because surface O atoms strongly withdraw electrons from adjacent Ru atoms, producing a pronounced $Ru^{\delta+(high)}–Ru^{\delta+(low)}$ charge gradient. This asymmetric Lewis acid–Lewis acid pair polarizes the O–H bond of phenol, stabilizes the formation of the phenoxide-like intermediate ($C_6H_5O^-$), and facilitates H transfer to the partially oxidized Ru site. The highly polarized Ru–O interface, therefore offers an electronically ideal platform for heterolytic O–H activation.

On the OH-rich surface ($Ru_{18}(OH)_{16}$), the O–H bond dissociation barrier increases to 0.28 eV. Although the surface is still partially oxidized, the pre-adsorbed OH groups partially neutralize the $Ru^{\delta+}–O^{\delta-}$ polarization that drives heterolytic activation. Moreover, the OH ligands occupy interfacial positions that otherwise would participate in charge separation, limiting the ability

of Ru–O pairs to stabilize the transition-state charges. Consequently, phenol O–H activation still occurs but with reduced efficiency compared to the O-rich surface.

On the OH-deficient surface ($Ru_{18}(OH)_5$), the barrier rises sharply to 0.59 eV. In the absence of interfacial oxygen ligands, the Ru cluster remains largely metallic, lacking sufficient charge heterogeneity to polarize the O–H bond. Without meaningful $Ru^{\delta+}$–$Ru^{\delta+}$ asymmetry, phenol O–H dissociation must proceed via a less favorable, weakly polarized pathway. This behavior highlights that metallic Ru alone is not intrinsically effective for phenolic O–H scission and must rely on oxidation-induced electronic perturbation to activate such bonds efficiently.

In summary, phenol O–H dissociation follows the order O-rich (0.17 eV) < OH-rich (0.28 eV) << OH-deficient (0.59 eV), directly mirroring the degree of surface charge polarization induced by oxygen ligands. The O-rich Ru/CNF surface provides the strongest $Ru^{\delta+}$–$O^{\delta-}$ pairing, enabling heterolytic O–H activation, whereas excessive hydroxylation dampens this effect and OH deficiency eliminates it entirely.

Supplementary note 2: hydrogenation of aromatic ring

The energy barriers for benzene-ring hydrogenation further underscore the strong promoting effect of surface polarity. The O-rich $Ru_{18}(OH)_8O_4$ catalyst exhibits the lowest hydrogenation barrier (0.50 eV), followed by the OH-rich $Ru_{18}(OH)_{16}$ surface (0.65 eV), whereas the OH-deficient $Ru_{18}(OH)_5$ catalyst displays a dramatically higher barrier (1.54 eV). The transition state structures on three catalysts are summarized in Table S6. These trends correlate directly with the strength of phenoxide binding and the degree of charge polarization induced at the Ru–O interface.

On the O-rich surface, phenol dissociation forms a strongly bound phenoxide species, $C_6H_5O\cdots Ru^{\delta+}–O^{\delta-}(OH^{\delta+})$, which anchors the aromatic ring in a polarized geometry. In this charge-separated configuration, the two ortho carbons adjacent to the ipso C–O bond become electron deficient, because electron withdrawal by the $O^{\delta-}$ ligand propagates through the phenoxide π system and alters the electron density across the ring. This increases the electrophilicity of the ortho carbon, making it more susceptible to nucleophilic hydride attack and thereby lowering the hydrogenation barrier.

There are two factors that work together to reduce the benzene-ring hydrogenation barrier on the O-rich surface. (1) enhanced ring polarization through $Ru^{\delta+}–O^{\delta-}$ charge separation: the strong electron-withdrawing effect of surface $O^{\delta-}$ produces a highly oxidized $Ru^{\delta+}$ center. When the phenoxide binds through O, the adjacent ortho carbon becomes electron poor, thus more reactive toward incoming hydride. This facilitates the first C-H addition step, which is typically rate-determine step in aromatic ring hydrogenation. (2) activation of hydride transfer through $Ru^{\delta+}$ sites: on the O-rich surface, the Ru atoms farther from the O-ligand remain less oxidized ($Ru^{\delta+}$) and retain higher electron density. Hydride bound to these $Ru^{\delta+}$ sites is therefore more nucleophilic.

In contrast, the OH-rich surface exhibits weaker Ru–O polarization and partial charge neutralization by surface hydroxyls, leading to a moderate barrier. The OH-deficient surface lacks the interfacial polarity required to activate either the aromatic ring or the Ru–H bond, resulting in the highest barrier.

Overall, the O-rich Ru/CNF catalyst lowers the aromatic hydrogenation barrier by combining an electrophilically polarized ortho carbon with a nucleophilic Ru-bound hydride, a cooperative effect absent on OH-rich and OH-deficient surfaces.

Supplementary note 3:

The two competing reaction pathways naturally lead to distinct product distributions: direct deoxygenation primarily yields aromatic hydrocarbons, whereas the hydrogenation-deoxygenation pathways produce saturated cycloalkanes. The fine chemical state of Ru plays a decisive role in directing these pathways. Unlike Al_2O_3 supports, where hydroxyl species remain largely immobile, or activated carbon that is predominantly inert, the CNF support facilitates dynamic surface restructuring, enabling partial oxidation of Ru and the formation of bifunctional RuO_x/Ru ensembles.

The catalytic performance trends can be directly correlated with the surface oxidation states of the Ru/CNF catalysts and the resulting electronic structure at the Ru/RuO_x interface. The oxidized CNF support is intrinsically OH-rich, and both MD simulations and XPS characterization confirm that thermal treatment at 573 K drives the migration of surface OH groups toward the Ru cluster, forming a stable Ru/RuO_x interfacial structure. This partial oxidation yields an O-decorated Ru surface in which adjacent Ru atoms acquire different degrees of electron deficiency. Such a mixed-valence $Ru^{\delta_{+}+}$ –$Ru^{\delta^{+}}$ arrangement, which polarize both adsorbed molecules and the Ru-H bond.

The presence of gradient interfacial active sites significantly enhances catalytic reactivity. First, polarization at the Ru/RuO_x interface lowers the energy barrier for H_2 activation by enabling heterolytic H-H cleavage across charge-imbalanced Ru sites, as demonstrated in the DFT calculations. Second, the same polarized environment strongly activates phenol: the O-H bond dissociation, aromatic ring polarization, and subsequent hydrogenation all proceed with substantially reduced barriers on the O-rich surface. This explains why Ru/CNF exhibits dramatically higher HDO activity than OH-deficient samples or Ru supported on AC or Al_2O_3.

Importantly, the strong phenoxide binding and ring polarization created at the Ru/RuO_x interface selectively promote aromatic ring hydrogenation over direct deoxygenation, steering the reaction pathways toward cyclohexanol and subsequently to cycloalkanes. This accounts for the remarkably high selectivity to C_6-C_9 cycloalkanes observed for Ru/CNF and Ru/CNF-700, in stark contrast to catalysts lacking sufficient surface polarity, which instead yield predominantly aromatic products.

In summary, even for the simplified model system such as phenol hydrogenation, it involves hydrogen activation, phenol adsorption and hydrogenation, and C-O activation and cleavage. In fact, the reactions require a large ensemble of active sites instead of well-defined single or dual active sites. The active sites ensemble is highly asymmetric in terms of Ru charge, and the active sites are highly cooperative, where the different sites play different roles in the catalytic cycle. The hydrogenation activation occurs on the $Ru^{\delta+ +}$-$Ru^{\delta+}$ partially charged pair, where ++ indicates a higher charge. The pair can be described as a Lewis acid pair within the ensemble of $O^{\delta-}$-$Ru^{\delta++}$-$Ru^{\delta+}$, leading to heterolytic association to form H-$Ru^{\delta++}$ and H-$Ru^{\delta+}$. The asymmetric charge distribution significantly lowered the energy barrier of H_2 dissociation. The adsorption of phenol requires a large ensemble of active sites, where the aromatic ring is adsorbed on Ru surface via π electron interaction. The aromatic ring hydrogenation involves the reaction between the H-$Ru^{\delta++}$ and adsorbed phenol.

Collectively, the DFT results, using phenol as a model compound, support a mechanistic hypothesis in which surface oxygen functionalities regulate key steps within the HDO reaction network by modulating the electronic structure of the interfacial Ru/RuO_x active site. This modulation influences hydrogen activation capability and the polarization of C–O bonds in phenolic intermediates. The model suggests that an O-rich Ru/CNF surface combines electron-deficient Ru sites, which facilitate H_2 activation, with oxygenated sites that strongly bind and polarize -OH and C–O moieties. This cooperative interaction appears to lower the calculated

barriers for O–H cleavage and subsequent hydrogenation steps, providing a plausible electronic-structure rationale for the enhanced HDO performance observed experimentally.

While these computational insights outline a structure–activity relationship linking surface oxidation to catalytic function and offer a framework for designing Ru-based catalysts via engineered Ru/RuO_x interfaces, the calculated energy landscapes describe intrinsic pathways only for the simplified model reaction. Nevertheless, they help rationalize the experimentally observed structure–performance relationship in lignin conversion.

Supplementary References

1 Bai, Y. *et al.* Catalytic depolymerization of a lignin-rich corncob residue into aromatics in supercritical ethanol over an alumina-supported NiMo alloy catalyst. *Energy & Fuels* **33**, 8657-8665 (2019).

2 Perdew, J. P. *et al.* Restoring the Density-Gradient Expansion for Exchange in Solids and Surfaces. *Phys. Rev. Lett.* **100**, 136406 (2008).

3 Kresse, G. & Furthmüller, J. Efficient iterative schemes for ab initio total-energy calculations using a plane-wave basis set. *Phys Rev B* **54**, 11169-11186 (1996).

4 Kresse, G. & Joubert, D. From ultrasoft pseudopotentials to the projector augmented-wave method. *Phys Rev B* **59**, 1758-1775 (1999).

5 Alavi, A., Hu, P., Deutsch, T., Silvestrelli, P. L. & Hutter, J. CO Oxidation on Pt(111): An Ab Initio Density Functional Theory Study. *Phys. Rev. Lett.* **80**, 3650-3653 (1998).

6 Wang, D., Sheng, T., Chen, J., Wang, H.-F. & Hu, P. Identifying the key obstacle in photocatalytic oxygen evolution on rutile TiO2. *Nat. Catal.* **1**, 291-299 (2018).

7 Yuan, H., Chen, J., Guo, Y., Wang, H. & Hu, P. Insight into the Superior Catalytic Activity of MnO2 for Low-Content NO Oxidation at Room Temperature. *J. Phys. Chem. C* **122**, 25365-25373 (2018).

8 Yuan, H., Chen, J., Wang, H. & Hu, P. Activity Trend for Low-Concentration NO Oxidation at Room Temperature on Rutile-Type Metal Oxides. *ACS Catal.* **8**, 10864-10870 (2018).

9 Wang, D., Wang, H. & Hu, P. Identifying the distinct features of geometric structures for hole trapping to generate radicals on rutile TiO2(110) in photooxidation using density functional theory calculations with hybrid functional. *Phys. Chem. Chem. Phys.* **17**, 1549-1555 (2015).

10 Chen, Z. *et al.* Understanding the Dual Active Sites of the FeO/Pt(111) Interface and Reaction Kinetics: Density Functional Theory Study on Methanol Oxidation to Formaldehyde. *ACS Catal.* **7**, 4281-4290 (2017).

11 Li, X. *et al.* One-Pot Conversion of Lignin into Naphthenes Catalyzed by a Heterogeneous Rhenium Oxide-Modified Iridium Compound. *ChemSusChem* **13**, 4409-4419 (2020).

12 Xia, Q. *et al.* Direct hydrodeoxygenation of raw woody biomass into liquid alkanes. *Nat. Commun.* **7**, 1-10 (2016).

13 Duan, H. *et al.* Hydrodeoxygenation of water-insoluble bio-oil to alkanes using a highly dispersed Pd–Mo catalyst. *Nat. Commun.* **8**, 591 (2017).

14 Shao, Y. *et al.* Selective production of arenes via direct lignin upgrading over a niobium-based catalyst. *Nat. Commun.* **8**, 16104 (2017).

15 Kong, J., He, M., Lercher, J. A. & Zhao, C. Direct production of naphthenes and paraffins from lignin. *Chem. Commun.* **51**, 17580-17583 (2015).

16 Wang, D. *et al.* Lignin Valorization: A Novel in Situ Catalytic Hydrogenolysis Method in Alkaline Aqueous Solution. *Energy & Fuels* **32**, 7643-7651 (2018).

17 Guo, T., Xia, Q., Shao, Y., Liu, X. & Wang, Y. Direct deoxygenation of lignin model compounds into aromatic hydrocarbons through hydrogen transfer reaction. *Applied Catalysis A: General* **547**, 30-36 (2017).

18 Kong, L., Liu, C., Gao, J., Wang, Y. & Dai, L. Efficient and controllable alcoholysis of Kraft lignin catalyzed by porous zeolite-supported nickel-copper catalyst. *Bioresource Technology* **276**, 310-317 (2019).
